# Substances of Natural Origin in Medicine: Plants vs. Cancer

**DOI:** 10.3390/cells12070986

**Published:** 2023-03-23

**Authors:** Adrianna Gielecińska, Mateusz Kciuk, Somdutt Mujwar, Ismail Celik, Damian Kołat, Żaneta Kałuzińska-Kołat, Renata Kontek

**Affiliations:** 1 Doctoral School of Exact and Natural Sciences, University of Lodz, 90-237 Lodz, Poland; 2 Department of Molecular Biotechnology and Genetics, University of Lodz, 90-237 Lodz, Poland; 3 Chitkara College of Pharmacy, Chitkara University, Rajpura 140401, Punjab, India; 4 Department of Pharmaceutical Chemistry, Faculty of Pharmacy, Erciyes University, Kayseri 38039, Turkey; 5 Department of Experimental Surgery, Faculty of Medicine, Medical University of Lodz, Narutowicza 60, 90-136 Lodz, Poland

**Keywords:** cancer, paclitaxel, irinotecan, betulinic acid, resveratrol, roburic acid

## Abstract

Continuous monitoring of the population’s health is the main method of learning about disease prevalence. National and international data draw attention to the persistently high rates of cancer incidence. This necessitates the intensification of efforts aimed at developing new, more effective chemotherapeutic and chemopreventive drugs. Plants represent an invaluable source of natural substances with versatile medicinal properties. Multidirectional activities exhibited by natural substances and their ability to modulate key signaling pathways, mainly related to cancer cell death, make these substances an important research direction. This review summarizes the information regarding plant-derived chemotherapeutic drugs, including their mechanisms of action, with a special focus on selected anti-cancer drugs (paclitaxel, irinotecan) approved in clinical practice. It also presents promising plant-based drug candidates currently being tested in clinical and preclinical trials (betulinic acid, resveratrol, and roburic acid).

## 1. Introduction

Despite the advances in modern medicine and pharmacology, cancer is a global problem, posing one of the greatest challenges of the 21st century. It is assumed that one in six deaths is due to cancer [1]. The late diagnosis affects the rate of progression of pathological changes and minimizes the chances of remission of the disease. It is predicted that the burden of cancer is likely to intensify over the next several years [2]. High incidence and mortality rates are causing scientific and research centers to intensify their efforts to reduce the cancer burden, through endeavors aimed at developing effective oncopharmaceuticals. For decades, substances of natural origin have been the main reservoir of therapeutic agents, playing a key role in clinical use and prevention [3,4]. Their extraordinary diversity makes them a rich collection of active compounds with high therapeutic potential. These substances are isolated from microorganisms and animal organisms, but special merit is given to compounds of plant origin [5,6,7]. Their therapeutic properties have been particularly appreciated in oncology, where it is estimated that about 60% of all oncopharmaceuticals available on the drug market have been designed based on plant compounds [8,9,10]. Anthracyclines, taxanes, and *vinca* alkaloids are just some of the groups of compounds commonly used as anti-cancer drugs [11,12,13]. Many other promising compounds have been tested, reported, and validated, but have not entered clinical practice. This is attributed to the poor water solubility, low stability, and bioavailability of natural compounds. Another obstacle is the isolation of compounds on an industrial scale, which also limits future production possibilities. Moreover, most of the research conducted in the mid-twentieth century was based on phenotypic screening, which did not focus on the specific mechanisms of action, but only on the effect caused by the substance [14]. This gave an incomplete depiction of the characteristics and therapeutic potential of the substances studied. As a result, many scientific research centers and pharmaceutical companies have reduced their work on natural compounds, thereby shifting their attention to chemically synthesized compounds [15]. Obtaining these compounds has proven to be faster and more efficient than extracting substances from natural raw materials.

Intensive technological and methodological development involves introducing information systems, developing computational methods, creating programs to study protein structures, and predicting both molecular targets and drug-target interactions. High-throughput experimental techniques that can be used to screen new chemical entities, combined with conceptual advancement, has led to the emergence of interdisciplinary fields of science, targeted at understanding the biological mechanisms and processes in living organisms. The available tools allow for the analysis of genes, genomes, protein expression, and signaling and metabolic pathways, but importantly, also allow for faster and more efficient study of chemical compounds and drug design. By using the achievements of nanotechnology in medicine as modern drug delivery systems, the so-called nanoparticles (liposomes, micelles, lipid nanoparticles, polymer nanoparticles, etc.) makes it possible to overcome the problem of low bioavailability of compounds, and also improves compounds’ solubility and metabolic rate [16,17,18]. The possibility of overcoming the previous difficulties related to physicochemical properties, substance availability, and the complexity of pharmacological effects, can realistically influence the transfer of tested compounds from research laboratories to widespread use in clinics. It justifies the recently observed trend of reinventing and researching natural substances, which, combined with modern tools, gives an opportunity to better understand their properties and potential therapeutic utility [19]. At the same time, their multifaceted mechanisms of action are being discovered, as well as their tendency to simultaneously affect multiple oncogenic signaling pathways by modulating the expression of their molecular targets, which translates into the ability of cells to proliferate, migrate, metastasize or undergo apoptotic cell death. Due to the complexity of their properties, natural compounds can be used directly or as chemotherapeutic adjuvants, supporting anti-cancer activity, overcoming therapy resistance in cancer cells, and supporting cells’ repair mechanisms [20,21]. In addition to their important therapeutic role, compounds of natural origin may be leading structures in the search and development of other pharmaceuticals. On their basis, modifications can be introduced and analogs with increased affinity to the target and higher selectivity to the enzyme isoforms can be synthesized. The selection of the leading structure and the synthesis of derivatives from natural compounds seems to be more effective when compared to the use of synthetic compounds. In the era of current efforts and opportunities, many natural compounds that are newly discovered or re-tested can be expected to reach the next stages of drug development and final clinical use. In this paper, we present an up-to-date review of both the best-studied and the new, potential plant compounds with anti-cancer properties.

## 2. Main Risk Factors

Cancer susceptibility often has a genetic basis. The disease can be triggered by a rare inherited gene mutation with high or low penetrance in carriers, and cause so-called familial syndromes, i.e., breast or ovarian cancer with breast cancer gene 1 (*BRCA1*) or breast cancer gene 2 (*BRCA2*) mutations, and Li-Fraumeni syndrome associated with rare tumor protein P53 (*TP53*) variants that lead to development of soft tissue sarcomas, breast cancer, colon cancer, gastric cancer, and brain tumors [22]. Diagnosis of hereditary cancer predisposition syndrome is based on pedigree analysis, clinical evaluation, and genetic testing. Alternatively, common genetic variants with the sporadic risk that do not have a clear hereditary syndrome may lead to cancer occurrence. Their role in shaping enzyme and protein activity is important in the context of mutations and DNA repair. In addition, the accumulation of low penetrance variants or susceptibility gene loci within target genes may affect pathways and mechanisms involved in cancer development [23]. 

Environmental etiological factors of cancer include poor eating habits, obesity, smoking, lack of physical activity, and exposure to air or water pollution, radiation, or infectious pathogens [24,25,26,27]. For instance, excess insulin levels, caused by high blood glucose often found in people with excessive body fat, will lead to lower insulin-like growth factor binding protein 3 (IGFBP-3) levels. Increased levels of unbound insulin-like growth factor-1 (IGF-1) promote cancer formation. Hyperinsulinemia also modifies the bioavailability of sex hormones, resulting in prolonged exposure to free estrogens and androgens, which, unopposed by hormones with anti-proliferative effects, can promote oncogenesis [27,28]. Cigarette smoke contains more than 50 carcinogens (e.g., nitrosamines and DNA methylating agents) that cause numerous types of DNA damage. About 60% of cases of malignant melanoma are caused by the genotoxic effects of UVA and UVB radiation, which is exacerbated not only by lack of protection from the sun’s rays but also by the fashion of using tanning beds. Most of the presented environmental risk factors are modifiable. Thus, it is possible to influence the burden of cancer through actions that contradict these factors. If the disease progresses despite prophylactic measures, systemic treatment with cytostatic drugs (chemotherapy), hormonally active drugs (hormone therapy), drugs that stimulate the immune system (immunotherapy), or those that target specific pathways and signaling pathways (targeted therapy) is implemented. 

## 3. Conventional Treatment

Chemotherapy remains the most common and effective method of cancer treatment. Its aim is to damage the genetic material of cancer cells. The presence of damage activates the DNA damage response pathway (DDR), which constitutes a detection, signaling, and repair system [29]. The DDR stimulates subsequent molecular mechanisms and triggers events leading to inhibition of the cell cycle progression and cell death [30]. The use of agents that induce DNA damage is a primer of the cancer treatment. Based on their mechanism of action, chemotherapeutics are divided into alkylating agents, antimetabolites, topoisomerase inhibitors, mitotic spindle inhibitors, and others [31,32]. These include substances of both natural (plant) and synthetic origin. 

Alkylating agents act by alkylating DNA, causing structural changes in DNA through the attachment of alkyl groups to DNA bases, leading to inappropriate base pairing, depurination of DNA molecules, and formation of inter- or intrastrand cross-links. The versatility of DNA damage caused by a specific agent depends mainly on the number of its reactive sites (mono or bifunctional agents). Monofunctional agents contain a single chemical grouping that modifies a single base in DNA molecule, while bifunctional agents contain two groups that can bind to different sites in DNA [33]. Damage prevents the process of replication and transcription from taking place. Alkylating agents currently used in chemotherapy include several groups of substances, including oxazsaphosphines (cyclophosphamide, ifosfamide), nitrogen iperities (melphalan, chlorambucil), platinum derivatives (cisplatin, oxaliplatin), nitrosourea derivatives (carmustine, lomustine), and triazenes (dacarbazine, temozolomide) [31,33]. 

Antimetabolites are chemical compounds that, due to their structural similarity to metabolites, can displace them from specific metabolic pathways, thereby disrupting their course. Purine or pyrimidine analogs (6-mercaptopurine, 5-fluorouracil) are incorporated into nucleic acids, disrupting their proper synthesis and the fidelity of the genetic material further transferred to progeny [34]. Some antifolates (methotrexate) interfere with the activity of enzymes, i.e., dihydrofolate reductase, which supply cells with tetrahydrofolate and carry mono-carbon groups that act as coenzymes in the synthesis of purines and pyrimidines and the metabolism of homocysteine, methionine, and other amino acids [35]. Among the best-studied antimetabolites are pyrimidine analogs 5-fluorouracil, tegafur, capecitabine, cytarabine, gemcitabine, azacitidine; purine analogs, including azathioprine, 6-mercaptopurine, thioguanine, cladribine, clofarabine, fludarabine); antifolates, including methotrexate, pralatrexate, pemetrexed; thymidylate synthase inhibitors (raltitrexed); ribonucleotide reductase inhibitors (hydroxyurea); and others [31,34,36]. 

Topoisomerases are a group of enzymes responsible for maintaining correct DNA topology during replication and transcription that pose a torsional threat to DNA molecules. Inhibition of their action causes fragmentation of the genetic material of cells through the formation of DNA strand breaks, which, if unrepaired, will lead to cell death [37]. One of the first known topoisomerase inhibitors was camptothecin, isolated from the plant *Camptotheca acuminata*. The formation of DNA-protein complexes at the ends of the DNA breaks prevents the restoration of the proper DNA structure. However, due to its high toxicity and poor solubility, the substance was withdrawn from clinical trials [10]. Based on the natural derivative, two semi-synthetic derivatives were created: irinotecan and topotecan. Other substances with inhibitory effects on topoisomerases include etoposide, teniposide, and anthracyclines [34]. 

**Figure 1:**
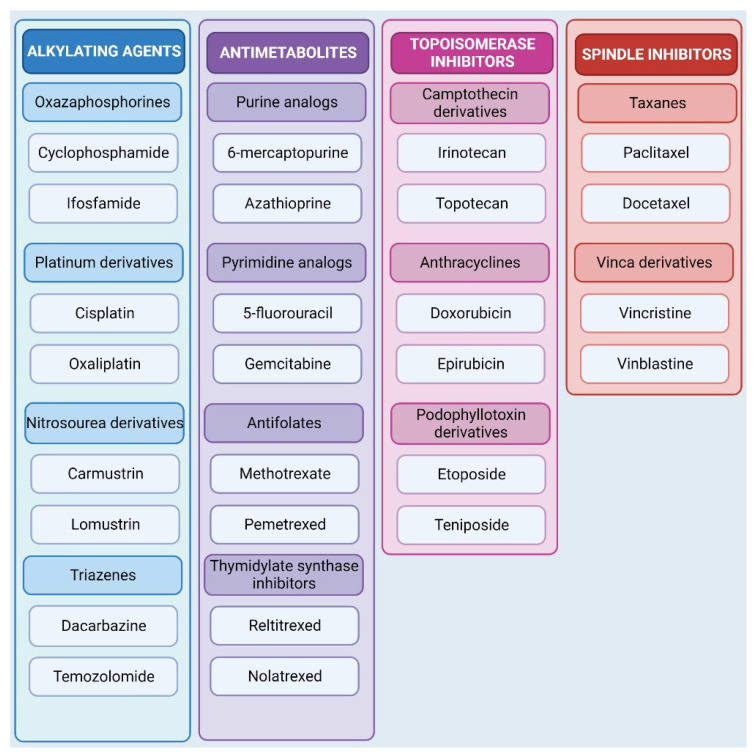
The main types of chemotherapeutics sorted by their mechanism of action. Among the most popular are alkylating compounds, antimetabolites, topoisomerase inhibitors, and mitotic spindle inhibitors. Created with BioRender.com.

Another effective approach in the fight against cancer cells is targeting cell division. Microtubules play a key role in the segregation of chromosomes during cell division. Structurally, microtubules consist of α-tubulin and β-tubulin, which are a network of protein filaments whose dynamics are responsible for the proper segregation of chromosomes [37,38]. *Catharanthus roseus* contains a broad variety of alkaloids, i.e., vincristine and vinblastine with proven anti-cancer activity. They are commonly used in lung and breast cancers or lymphomas due to their ability to inhibit microtubule movement or depolymerization, leading to disruption of their structure and arrest of cells in the M phase of the cell cycle [39]. However, their use is associated with high hepatotoxicity and neurotoxicity, which not only caused damage to internal organs, but also prevents the continuation of therapy. Due to the adverse side effects of the use of vincristine and vinblastine, their semi-synthetic analogs—vindesine and vinorelbine—have been created, which have a reduced toxicity profile [34]. Another group of compounds aimed at inhibiting mitosis are taxanes, which are isolated from the needles and bark of yew trees. The first drug developed from this group with anti-mitotic activity is paclitaxel. It prevents the reorganization of the cytoskeleton, thereby disrupting cellular activities and leading to its death [37]. As some types of cancer are resistant to paclitaxel, a semi-synthetic derivative called docetaxel has been created that has a similar chemical structure and action. A summary of the previously mentioned groups of compounds is shown in Figure 1.

Through their increased capacity for growth and proliferation, dedifferentiation, immortalization, metastasis, and overall devastating effects, cancer cells pose a threat to the entire body. Targeting the key features of cancer—limiting the growth and division of cancer cells—remains an effective method of fighting cancer. Therefore, the search for substances that act on different stages of the cell cycle and prevent its progression is one of the primers in anti-cancer drug discovery. Inhibition of this process results from the effect of drug molecules on diverse pathways and mechanisms, i.e., interference with transcription (actinomycin D), induction of free radical formation (bleomycin), and inhibition of growth factor signaling (imatinib), that ultimately induce cancer cell death [40,41,42]. 

However, the cytostatics currently used in clinical setting have non-selective effects and affect not only cancer cells but also normal cells, especially rapidly dividing cells and those with limited repair capacity [43,44]. Therefore, the characteristic side effects of received chemotherapy are alopecia, pruritic skin, hand–foot syndrome, anemia, myelosuppression, vomiting, diarrhea, inflammation, damage to hair follicle cells, gastrointestinal tract, blood, and oral mucosa. Chemotherapy also carries a high risk of drug adverse effects due to acute toxicity of oncopharmaceuticals, causing damage to the liver (hepatotoxicity), kidneys (nephrotoxicity), nerves (neurotoxicity), and lungs (pulmonary toxicity) [45,46]. Long-term drug use can have distant consequences on the patient’s subsequent life and health, causing infertility, hypogonadism, or metabolic syndrome [47]. Side effects vary depending on the treatment regimen adopted, including the type of chemotherapy used, route of administration, and frequency of dosage. Therefore, there is an urgent need to combine conventional chemotherapeutics with non-toxic adjuvant compounds that can contribute to a better response to therapy and mitigate side effects.

## 4. Natural Compounds

Plants are a rich source of natural substances with medicinal properties. Their use dates back to ancient times, and in modern times, they are an undeniable staple of medicine and pharmacology. Most secondary metabolites do not have an essential effect on plant growth; they play specific functions in the protection against pathogens and potential predators, or they may serve as signaling substances responsible for interaction with symbionts [48,49]. These compounds protect plants but can be toxic to other organisms. Unlike primary metabolites, there are many of them, and their activity is relatively poorly understood. They may have a complex biosynthetic pathway accompanied by complex regulation. The synthesis of these substances can be limited to a species or even individuals. Their specific properties justify their use in various fields of medicine, including oncology. More than half of the oncopharmaceuticals currently used clinically are of plant origin, and estimates suggest that up to 3000 plants may have anti-cancer properties [8,9,10]. At the same time, ample evidence is emerging on the role of plant compounds as inhibitors of key steps in oncogenesis and related processes [9,50]. This underscores their importance as potential preventive and therapeutic agents. 

The use of substances of natural origin is not without drawbacks. Some plant-based compounds, due to their high toxicity, have been withdrawn from further studies during clinical trials, or their use has been restricted because of the number of side effects, i.e., paclitaxel and vinblastine. Nevertheless, they are considered more biologically friendly than synthetic compounds. Another obstacle is low bioavailability and poor water solubility of these compounds, which significantly limit their use. Therefore, efforts are being made to develop semi-synthetic analogs with improved physicochemical parameters and higher efficacies compared with the precursor natural compounds [51]. However, natural compounds of plant origin do not have to serve as stand-alone drugs. They can also be used as supportive or auxiliary substances that contribute to the reduction of side effects and enhance safety and pharmacokinetic profiles. The toxic burden on the body can be reduced by replacing part of the dose of a standard chemotherapeutic agent with a natural substance with a specific effect [52]. Natural compounds constitute a base for the design and development of new pharmaceuticals. 

Among the secondary metabolites of plant origin with proven therapeutic properties, several can be distinguished, including alkaloids, terpenoids (diterpenes, triterpenes), and polyphenols, such as flavonoids and lignans. Some of them have established and documented anti-cancer activity, as discussed in this review.

### 4.1. Paclitaxel

Paclitaxel is undoubtedly one of the leading compounds with anti-cancer activity. Since the introduction of paclitaxel to the pharmaceutical market, under the name Taxol^®^, it has been consistently popular due to its efficacy in the treatment of many types of cancer.

Paclitaxel belongs to the group of tricyclic diterpenoids. Structurally, paclitaxel encompasses the taxane ring system composed of a 6,8,6-tricyclic condensed scaffold connected to a four-membered ring of oxetane. It occurs naturally in the bark and needles of the short-leaf yew *Taxus brevifolia*. However, due to low availability or ecological reasons, alternative options for obtaining paclitaxel are used, i.e., plant cell culture [53], extraction from genetically modified fungi [54], chemical synthesis, or semi-synthesis [55]. The multi-step natural synthesis of this compound involves the cooperation of a variety of enzymes and makes the paclitaxel biosynthesis pathway complex and difficult to harness for the production of the drug [56]. 

The mechanism of action of paclitaxel differs compared to most compounds belonging to the class of division spindle inhibitors. During the initial stages of mitosis (prophase), a karyokinetic spindle, composed of microtubules, is formed. These fiber-like structures are responsible for the migration of chromosomes to opposite poles of the cell. Their dynamic instability, i.e., the ability to rapidly shorten or lengthen, is associated with the binding and hydrolysis of guanosine-5′-triphosphate (GTP). In the free state, GTP-bound αβ-tubulin dimers are attached to the growing microtubule by polymerization [57]. If the process is slower than GTP hydrolysis, microtubules are broken down and shortened. This situation occurs in anaphase, when tubulin molecules depolymerize, leading to the shortening of spindle elements and the separation of chromosomes. Paclitaxel increases the percentage of folding tubulin subunits, stabilizing microtubules and preventing spindle breakdown [58]. In this way, it disrupts the normal course of mitosis. However, the pathway from stopping cell division to cell death has not been thoroughly elucidated. Prevention of mitosis can lead to cell death, but also to mitotic slippage that is understood as an exit from the cell cycle without chromosome segregation or without cytoplasm division [58]. The mitotic checkpoint plays a key role in the process of paclitaxel-mediated mitotic arrest as it is one of the main mechanisms of cell cycle control during division; activation of this process promotes the sensitivity of cells to the drug and is required for their elimination. Both cells with normal and weakened mitotic checkpoints show similar sensitivity to the drug [59,60]; some theorized that cell fate may also depend on the length of time of mitosis arrest, meaning that the consequences should be the same for all or most cells. When attempts were made to verify this view, however, its credibility was shattered, and sisters cells were shown to have a different fate. It was therefore assumed that the response of cells to mitosis arrest is stochastic [61]. 

During numerous studies on paclitaxel, it was observed that, at certain concentrations, the compound induced the formation of multipolar spindles [62,63]. Cells arrested in metaphase tended to have bipolar spindles, and chromosomes aligned in the equatorial plane assumed a typical position. However, when using clinically relevant intratumor concentrations, paclitaxel did not lead to mitotic arrest. Instead, it caused cell death due to the abnormal segregation of multipolar spindles [64]. 

Currently, paclitaxel is one of the most potent anti-cancer drugs, mainly used as a first-line treatment for breast, ovarian, gastric, and non-small cell lung cancer [65]. It is also used in the treatment of other conditions, i.e., psoriasis [66], and acts as a neurotoxin inhibitor [67]. However, its utility is limited due to poor water solubility, the development of multidrug resistance (MDR) in the treated cells, or clinical neurotoxicity [68,69]. This has prompted the development of paclitaxel analogs that have a similar structure but improved pharmacokinetic parameters and reduced side effects. Various modifications of paclitaxel structure resulted in the introduction of docetaxel and cabazitaxel in clinical settings [70], and further led to the development of the paclitaxel liposomes, which involves encapsulating the drug in a nanocarrier to improve its distribution and solubility, as well as reducing the drug’s toxicity [65]. The generation of nab-paclitaxel, a nanoparticle drug bound to albumin, allowed increases in drug solubility and reduction of post-therapy neuropathy symptoms [71]. 

The effect that conventional chemotherapy has on the body’s immune system is currently gaining more and more attention. This is because the majority of studies examining the effectiveness of such drugs against cancer cells are carried out in vitro or on immunocompromised animals, and they do not include immunological follow-ups. In contrast, chemotherapy can provide the foundation for the immune system mobilization and reorganization, providing a tumor-targeted immune environment [72,73]. Paclitaxel and other compounds in the mitotic spindle inhibitor group have been shown to have immunomodulatory properties. Their effect on the stimulation of an adaptive anti-tumor immune response is manifested by the promotion of (a) the ability of cells to present antigens, (b) the activation of T and B lymphocytes, (c) the increase in the number and activity of natural killer effector cells, and (d) the production of antibodies and active substances, i.e., tumor necrosis factor α (TNF-α) and interleukins (IL-6, IL-8, and IL-12) [56]. Paclitaxel’s effects on the immune system were studied in C57BL/6 mice with Lewis lung cancer (3LL) [72]. Paclitaxel was shown to selectively reduce the number of regulatory T cells (Treg cells), impairing their ability to produce pro-inflammatory cytokines without affecting CD4+Foxp3- effector T cells (Teff) [72]. Treg cells are considered key players in immune suppression, capable of blocking the immune response, inhibiting T cell proliferation, and maintaining a state of immune tolerance. However, in neoplastic diseases, they show enhanced immunosuppressive activity, facilitating tumor growth. Therefore, reducing their engagement may provide a more tumor-oriented immune microenvironment. In addition, paclitaxel reduces the production of transforming growth factor β (TGF-β), which in advanced tumors, promotes epithelial–mesenchymal transition (EMT), angiogenesis, and immunosuppression [56,74,75]. Analyses of peripheral blood samples from patients with advanced non-small cell lung cancer (NSCLC) showed that paclitaxel treatment increases the percentage of Teff cells, and decreases Treg cells levels, but did not affect the number of CD8+ T cells and CD19+ B cells [76]. At the same time, an increase in the number of memory T cells, which are the basis for the development of long-term immune memory, was also noted; this may be a guarantee of sustained remission of the disease. Chemotherapy with paclitaxel in combination with carboplatin is commonly used to treat melanoma. Both drugs can stimulate innate and acquired immune responses [56]. The anti-mitotic effect of paclitaxel promotes apoptotic death of tumor cells, accelerating the release of antigens and their presentation to immune cells. When used in this scheme, carboplatin can reduce the activity of programmed death receptor-ligand 2 (PD-L2), expressed in cancer cells, which plays a key role in their ability to evade the immune system by inhibiting T cells [74]. Blocking the interaction of the programmed death receptor (PD-1) with its ligand PD-L2 increases the detection of cancer cells, preventing them from escaping the anti-tumor immune response. A number of clinical trials are currently underway to estimate the effects of paclitaxel–carboplatin in combination with various cytostatics and/or monoclonal antibodies for the treatment of lung cancer and other cancers [77]. Examples of preclinical studies with paclitaxel and its derivatives and ongoing clinical trials are presented in Table 1.

### 4.2. Irinotecan

Irinotecan is one of the most popular compounds in the topoisomerase inhibitor group used in oncotherapy. Although it is a semi-synthetic compound, it was developed based on a natural alkaloid, camptothecin, which showed significant anti-cancer activity, but was withdrawn from clinical trials due to its high toxicity and poor water solubility [37]. Many analogs have been developed based on this natural compound, including topotecan and irinotecan [81]. Of all the derivatives known to date, irinotecan (also known as CPT-11) is believed to have the highest therapeutic efficacy, and there are high hopes for its usefulness. 

Irinotecan is a pentacyclic compound with an embedded bis-piperidine side chain [82]. It can undergo structural changes when exposed to the pH of the cellular environment. One of the key elements of irinotecan is the lactone ring, responsible for its metabolic activity and anti-cancer properties. Opening of the ring can lead to the inactivation of the compound because it is converted to a carboxylate that is impermeable through cell membranes [37]. Irinotecan exists in the form of a pro-drug, a substance that requires metabolic activation to exert biological activity. As a result of the hydrolysis of the compound by hepatic enzymes of the carboxylesterase group, irinotecan is converted to the active and highly toxic metabolite 7-ethyl-10-hydroxycamptothecin (SN38) [83]. Although the bioactivation process occurs rapidly, a significant portion of irinotecan is excreted without being pre-metabolized. The resulting SN38 is transported to the liver, where it is inactivated and converted to SN38-glucuronide (SN38-G) by the microsomal enzyme uridine 5′-diphosphate-glucuronosyltransferase (UGT) [84]. This step is designed to detoxify the metabolite and facilitate its elimination from the body. The metabolites of irinotecan are excreted mainly in the feces, except for SN38-G, which is excreted with the bile [85]. In the final step, SN38-G is transported to the intestine, where it can be reactivated by bacterial β-glucuronidase (βG). Local reactivation of SN38 results in its reabsorption [86].

The main target If irinotecan’s action is the nuclear enzyme topoisomerase I, which is responsible for maintaining the proper topology of DNA during the replication and transcription of genetic material [87,88]. Its task is to alleviate torsional tensions formed on the DNA molecule by the movement of multiprotein replication or transcription complexes along the strand. As proteins move along the strand, they unravel the double helix, causing buildup of positive and negative supercoils [89]. Supercoils negatively affect the availability of DNA for transcription factors and prolong the duration of the process. In addition, they disrupt the correct shape of DNA, which is unable to rotate around its axis. Therefore, enzymes are needed to enable the regulation of torsions exerted on the DNA molecule. The resolution of torsional stress is carried by topoisomerase enzymes, which are responsible for the addition of supercoils and their removal (DNA relaxation). Under physiological conditions, topoisomerase I cleaves a single ester bond in a DNA strand by covalently binding a tyrosyl group in the active site of the enzyme to a phosphoryl group of DNA molecule [90]. This is a fully controlled and reversible transesterification reaction, resulting in DNA relaxation [82]. The topoisomerase-DNA crosslink is hydrolyzed as soon as the supercoil is removed, and the DNA strands are ligated again. The attachment of irinotecan to the DNA-bound topoisomerase blocks the dissociation of the enzyme from the DNA molecule, leading to the exposition of the ternary complex for collision with replication forks followed by the formation of double-strand breaks (DSBs) in the DNA molecule. The resulting damage is detected by DDR, which forms a signaling cascade and activates subsequent molecular mechanisms [29], such as activation of cell cycle checkpoints, DNA repair, or apoptosis [91]. 

Topoisomerase I remains the main but not the only target of action of irinotecan and its metabolites. Other topoisomerase-independent interactions of SN38 with protein factors were reported. For example, the active metabolite of irinotecan has been shown to inhibit the DNA binding activity of human far upstream element binding protein 1 (FUBP1) [92]. The FUBP1 protein is important for the normal development of the nervous system and lungs [93,94]. Its overexpression is found in many cancers, where it plays an anti-apoptotic role, promoting the expansion of cancer cells. The interaction of SN38 with FUBP1 prevents the oncoprotein from binding to molecular targets. Other proposed targets include the mouse homolog of double minute 2 (MDM2) that works as the E3 ligase of the tumor suppressor *TP53* and the anti-apoptotic B-cell lymphoma-extra large (BCL-xL) in human hepatocellular carcinoma cells. These interactions result in the reduction in cell proliferation and cell-cycle arrest in the G2/M phase [92]. Takeba et al. demonstrated that SN38 promotes apoptosis in a *TP53*-dependent manner [95]. However, apoptosis is not the only cellular response to irinotecan treatment. Cells exposed to SN-38 exhibit characteristic features of senescent cells including growth arrest, increased size and granularity, polyploidization or accumulation of P21 and cyclin D1 proteins [96]. These and other findings demonstrate that camptothecin derivatives are still an under-explored source of therapeutic agents with multidirectional effects.

For more than 2 decades, irinotecan has been used to treat colorectal cancer [97], lung cancer [98], stomach cancer [99], ovarian cancer [100], and others. Approved by the US Federal Drug Administration (FDA) under the name Campto^®^, it is used both as monotherapy and in combination with other chemotherapeutics. Combinations with kinase inhibitors [101,102], platinum-based drugs [103], or immunotherapy [104] are also common. Efforts are also undertaken to improve drug stability and reduce gastrointestinal toxicity by introducing prophylactic agents that inhibit SN38 reabsorption (alkylating drugs, plant extracts, probiotics), as well as nanoparticles, peptides, and carbohydrates with the goal of improving the clinical utility of the drug. Oncoral^®^ has recently been developed as an enteric-coated tablet that is released in the duodenum, improving tolerance for the drug. In addition, liposomal formulations of irinotecan are also being introduced to protect against structural changes and chemical degradation and to improve the cytotoxic effect of irinotecan [105,106]. Improving quality and prolonging patient survival are overarching goals in efforts to develop effective formulations based on irinotecan and other camptothecin derivatives. Examples of preclinical studies with irinotecan and ongoing clinical trials are presented in Table 2.

### 4.3. Betulinic Acid

Betulinic acid (BA) is a pentacyclic compound that belongs to the triterpenoid group [110]. It occurs naturally in many plant species, including birch, eucalyptus, and plane trees. It exhibits a broad spectrum of biological properties, including anti-inflammatory and anti-cancer activities, making it an interesting candidate for pharmacological utility. Several of the BA derivatives known to date have entered clinical trials (NCT00701987, NCT00346502, www.clinicaltrials.gov).

The main method of obtaining betulinic acid is extraction from plant materials, with bark being the most commonly used material, and leaves and stems being less common. However, unlike betulin, the content of betulinic acid in extracts is low, usually about 4–12% [111]. These amounts are not sufficient to cover the market demand and allow free large-scale research. In addition, methods of sublimation, ultrasonic extraction, or extraction with organic solvents are not very environmentally friendly and involve the generation of hazardous waste [112,113]. Therefore, efforts have focused on other methods of obtaining BA, i.e., chemical synthesis. Since betulin is a natural precursor of betulinic acid, it is the primary substrate for its synthesis [111]. One method of BA synthesis involves the oxidation reaction of betulin using the Jones reagent, followed by the reduction of the resulting betulinic acid [114] or the chemoselective oxidation of betulin’s hydroxyl group directly to a carboxyl group at the C17 position [115]. The described BA synthesis reactions are time-consuming, and the reagents used in them are both toxic and expensive. Other techniques include biotransformation catalyzed by the fungi *Aspergillus foetidus* ZU-G1, *Aspergillus oryzae* [116], *Armillaria luteovirens Sacc* ZJUQH100-6 [117], and the expression of the compound in transgenic yeast [118]. Similar to other methods, biotransformation processes and genetic engineering tools have limitations and drawbacks, including the deep understanding of the betulinic acid biosynthetic pathways. Despite these facts, knowledge regarding its activity is steadily increasing. 

**Figure 2:**
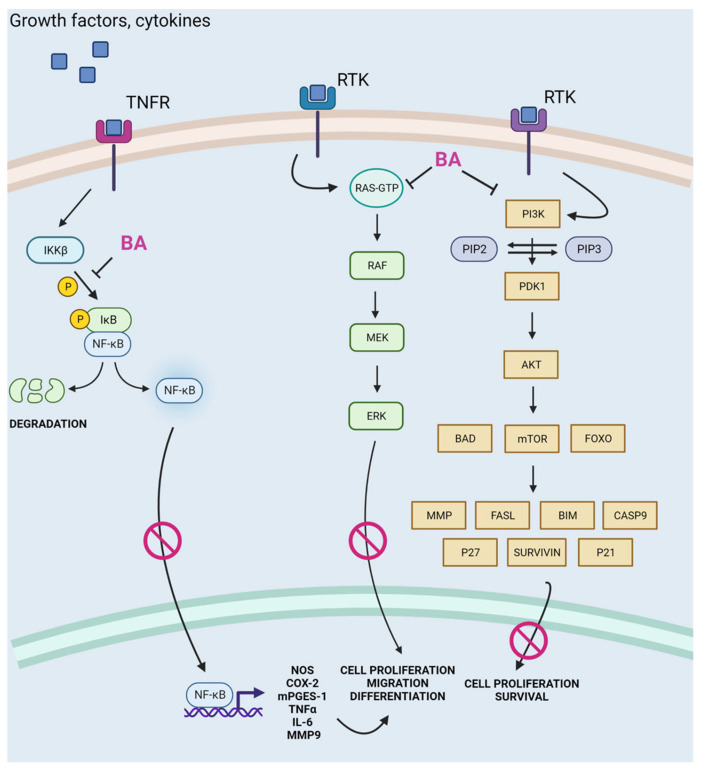
The activation of nuclear factor kappa-light-chain-enhancer of activated B cells (NF-κB) and the extracellular signal-regulated kinase (ERK)/mitogen-activated protein kinase (MAPK) and phosphoinositide 3-kinase (PI3K)/protein kinase B (AKT) pathways that support the development of the tumor and the pro-inflammatory cellular environment. Phosphorylation of B-cells inhibitor (IκB) by IκB kinase (IKKβ) as a result of cellular signals (growth factors, cytokines) received by receptor (tumor necrosis factor receptors (TNFR)), results in heterodimer disconnection, release of NF-κB, and degradation of IκB complex component. The released NF-κB translocates from the cytoplasm to the nucleus, where it acts as a transcription factor responsible for the expression of proteins involved in cell proliferation, migration, and inflammation. The activation of receptor tyrosine kinases (RTKs) initiates activation of ERK/MAPK and PI3K/AKT pathways, promoting survival, migration, and invasion of cancer cells. Binding of the ligand to the RTKs causes the “turning on” of the rat sarcoma virus (RAS) protein through GDP-to-GTP exchange, which activates the RAF proto-oncogene serine/threonine-protein kinase (RAF), mitogen-activated protein kinase (MEK) and ERK kinase, leading to the growth of cancer cells and supporting the transcription of pro-inflammatory factors. Similarly, PI3K is activated via RTKs, which catalyze the phosphorylation of phosphatidylinositol 4,5-biphosphate (PIP2) to phosphatidylinositol (3,4,5)-triphosphate (PIP3). As a result of the interaction of PIP3 and phosphatidylinositol kinase (PDK-1), AKT is activated. Being a serine-threonine kinase, AKT phosphorylates numerous proteins involved in the regulation of cell proliferation, growth, and survival. The key substrates for AKT are the mammalian target of rapamycin (mTOR) and the pro-apoptotic factor BCL-2-associated death promoter (BAD), which are capable of inhibiting apoptosis, and nuclear transcription factors fork head box O (FOXO). The use of betulinic acid (BA) prevents the phosphorylation of IκB, and thus, the release and translocation of NF-κB into the nucleus. As a consequence, the expression of inflammatory mediators and enzymes is not induced. BA also inhibits the activation of ERK/MAPK and PI3K/AKT signaling pathways, suppressing mechanisms oriented toward tumor growth and development. Created with BioRender.com.

Compounds belonging to the triterpene group often exhibit anti-diabetic and anti-inflammatory properties. The anti-inflammatory activity of the compounds is particularly important in the context of cancer development. Chronic inflammation leads to the formation of an inflammatory tumor microenvironment (TME) that is conducive to escape from the supervision of the immune system and promoting progression as well as metastasis [119]. The microenvironment is created by cells of the immune system, i.e., macrophages, B lymphocytes, subpopulations of T lymphocytes, dendritic cells, and natural killer (NK) cells, which act against cancer. The response generated by them, however, can be effectively inhibited by immunosuppressive cells (Tregs cells, type 2 macrophages), which drive the state of chronic inflammation and pro-angiogenic environment [120]. The constant presence of immune system cells and the proteolytic enzymes they produce can start to work in favor of the tumor, promoting survival, angiogenesis, and impairment of the function of anti-cancer lymphocyte populations. The anti-inflammatory and anti-diabetic properties of betulinic acid have been confirmed in vitro and in vivo. In a study by Khataylou et al., mice with autoimmune diabetes treated with BA exhibited a decrease in the inflammatory cytokines IL-1 and TNF-α in the serum and kidneys, with an increase in anti-inflammatory cytokines IL-10, C-peptide, and insulin, as well as an improvement in the histopathological changes of the kidneys. Betulinic acid may thus show efficacy in the treatment of inflammatory and autoimmune diseases [121]. In a mouse model of endotoxemia, the administration of BA protected mice from a lethal dose of lipopolysaccharide (LPS) [122]. It also caused an increase in IL-10 levels, which inhibited LPS-induced TNF-α release. In in vitro experiments, the same authors showed that BA treatment inhibited TNF-α and nitric oxide (NO) levels in LPS-activated macrophages, accompanied by an increase in IL-10 production [122]. The nuclear factor kappa-light-chain-enhancer of activated B cells (NF-κB) is one of the main factors responsible for the regulation of gene expression and the production of proteins associated with proliferation, inflammation, tumorigenesis, and cell death. It is present in the cytosol in an inactive state, with the nuclear factor of kappa light polypeptide gene enhancer in B-cells inhibitor (IκB) inhibitory protein [123]. As a result of incoming stimuli (including TNF family ligands), the IκB Kinase (IKKβ) is activated; this phosphorylates IκB and leads to its ubiquitination and degradation [123]. The released NF-κB moves to the nucleus, where it binds to DNA and induces the expression of pro-inflammatory factors [124]. Viji et al. showed that inhibition of IκBα by betulinic acid prevents nuclear translocation of NF-κB and, therefore, up-regulation of pro-inflammatory factors [125]. Furthermore, by inhibiting the cell signaling cascade of the NF-κB, extracellular signal-regulated kinase (ERK), mitogen-activated protein kinase (MAPK), and protein kinase B (AKT) pathways, betulinic acid contributes to the inhibition of the synthesis of critical pro-inflammatory mediators and enzymes, i.e., cyclooxygenase 2 (COX-2) and pro-inflammatory prostaglandin E2 (PGE2). Blockage of the ERK/Mitogen-activated protein kinase (MEK) signaling pathway was also observed, as well as a decrease in tumor cell migration and invasion [126] (Figure 2).

**Figure 3:**
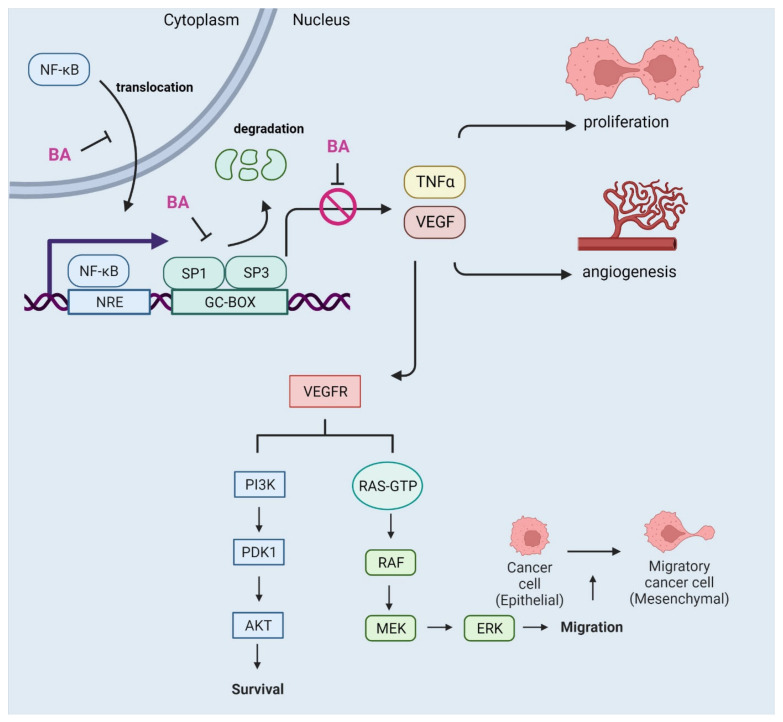
Tumor necrosis factor α (TNF-α) and vascular endothelial growth factor (VEGF) with pro-angiogenic function are overexpressed in tumors depending on transcription factors, i.e., transcription factor Sp1 (SP1) and transcription factor Sp3 (SP3). They are supported by the nuclear factor kappa-light-chain-enhancer of activated B cells (NF-κB), which bind to specific sequences known as NF-κB response elements (NRE) and GC-BOXes. The presence of VEGF in the cell enhances the activation of the rat sarcoma virus-GTP (RAS-GTP)/RAF proto-oncogene serine/threonine-protein kinase (RAF)/mitogen-activated protein kinase (MEK)/extracellular signal-regulated kinase (ERK) and phosphoinositide 3-kinase (PI3K)/phosphatidylinositol kinase (PDK-1)/protein kinase B (AKT) pathways, promoting invasive potential and tumor metastases. Betulinic acid (BA) prevents the translocation of NF-κB to the nucleus and promotes the degradation of SP1 and SP3, down-regulating VEGF. Created with BioRender.com.

Untreated chronic inflammation can lead to the development of cancer. Numerous studies have confirmed that BA exhibits not only anti-inflammatory but also anti-cancer properties. It acts in multiple ways, including topoisomerases inhibition (anti-proliferative activity) and suppression of angiogenesis, but its ability to induce the intrinsic apoptotic pathway is the major mechanism of BA action [111]. Since its anti-cancer properties in melanoma were described in 1995 [127], there have been regular reports of its use in other types of cancer. Lee et al. reported that BA induced apoptosis in A2780 ovarian cancer cells by Increasing the expression of caspases -3, -8, -9, and proapoptotic BCL-2-associated X protein (BAX) protein [128]. Treatment of BA ovarian cancer cells followed by Hoechst 33342 staining showed bright blue condensed nuclear chromatin, which is a morphological feature typical of apoptotic cells, suggesting that betulinic acid induces this type of cell death. Luo et al. showed that BA inhibits topoisomerases and decreases cyclin expression, leading to cell cycle arrest and apoptotic death [129]. Betulinic acid inhibits the binding of NF-κB to specific sequences of NF-κB response elements (NRE) and proteasome-dependent degradation of transcription factors, i.e., transcription factor Sp1 (SP1), transcription factor Sp3 (SP3), and down-regulation of vascular endothelial growth factor (VEGF) expression. SP1 and SP3 are transcription factors involved in chromatin remodeling; depending on transcript modification, by binding to GC-BOXes sequences in promoter regions, they may act as activators or repressors of gene expression of numerous of genes, including oncogenes and tumor suppressors as well as proteins involved in cell cycle regulation, damage response, or apoptosis [130]. Similarly, overexpression of VEGF in tumor cells correlates with an increase in tumor aggressiveness due to its potent pro-angiogenic functions. By activating various signaling pathways, i.e., phosphoinositide 3-kinase (PI3K)/AKT and MAPK/ERK, VEGF plays role in the chemotaxis and migration of cancer cells, and also stimulates the secretion of pro-inflammatory cytokines (TNF-α, IL-1β, IL-6) and proteolytic enzymes (matrix metalloproteinase-9 (MMP-9)) [131] (Figure 3). MMP-9 has a proteolytic cleavage activity for the extracellular matrix (ECM) enabling its remodeling and increasing the metastatic potential of cells. Degradation of basement membrane components (i.e., type IV collagen) by MMP-9 promotes tumor invasion and metastasis [131]. It has been observed that treatment of cancer cells with BA causes a decrease in the expression of MMPs, indicating that BA may be a protective factor in cancer progression [127]. As a consequence of BA treatment, human gastric cancer MGC-803 cells showed changes in mitochondrial membrane permeability (MiMP) with the cytochrome C release and activation of caspase -3 and -9 enzymes and nuclear DNA degradation [132]. These changes indicated the induction of death via the mitochondrial pathway of apoptosis. 

**Figure 4:**
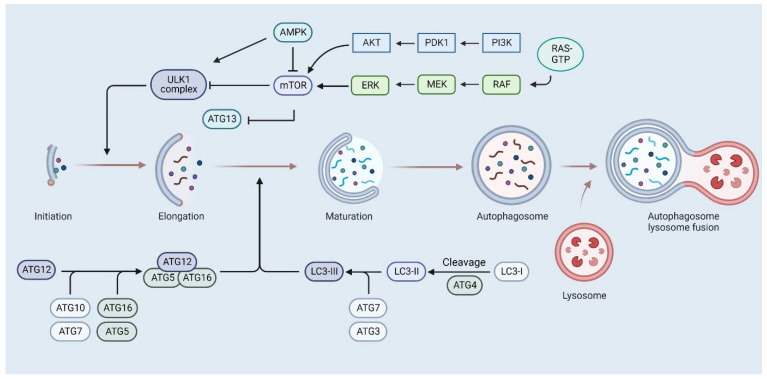
The process of autophagolysosome formation. Stimulated by the mitogen-activated protein kinase (MAPK) and protein kinase B (AKT) pathways, mammalian target of rapamycin (mTOR) and 5’ AMP-activated protein kinase (AMPK) regulate autophagy by interacting with Unc-51 like autophagy activating kinase (ULK1) complex and autophagy-related protein 13. ULK1, activated by AMPK, initiates autophagosome formation from intracellular membranes and its elongation. Then the autophagosome enters the elongation phase. Under the influence of the ATG3, ATG4 and ATG7 proteins, protein light chain (LC3) protein is converted to the LC3-I, LC3-II and LC3-III forms. The autophagy-related proteins (ATGs) (like ATG5, ATG7, ATG10, ATG12, ATG16) together with LC3-III contribute to the maturation of autophagosome that, through fusion with the lysosome, forms an autophagolysosome, which is a structure capable of enzymatic digestion of proteins. Created with BioRender.com.

Apoptosis is reportedly not the only BA-initiated cell death-related process. Autophagy is a catabolic process involving the massive degradation of cytoplasm and organelles through fusion with lysosomes [133]. Although the process physiologically mediates cell survival under stress conditions, it has been suggested that it may also be exploited to specifically target cancer cells [133]. Several different types of autophagy involve complex pathways and multiple protein factors. Among the best-studied proteins that trigger autophagy are mammalian target of rapamycin complex 1 (mTORC1) and 5′ AMP-activated protein kinase (AMPK). mTORC1 is a protein complex composed of mTOR kinase and mTOR associated protein LST8 homolog (MLST8) [134]. Acting as a negative regulator, mTORC1 phosphorylates proteins essential for the occurrence of autophagy, i.e., autophagy-associated protein 13 (ATG13) and Unc-51 like autophagy activating kinase (ULK1), inhibit their activity [133,134,135]. The role of AMPK is to promote autophagy by inducing ULK1 [134]. Inactivation of mTORC1 combined with simultaneous activation of AMPK drives the formation of a phagophore structure from intracellular membranes. The formation of the phagophore membrane is mediated by autophagy-associated protein 5 (ATG5), autophagy-associated protein 12 (ATG12), and autophagy-associated protein 16 (ATG16). The vesicular protein sorting kinase 34 (VPS34), in complex with beclin 1, stimulates the production of PIP3, which is necessary for phagophore elongation and recruitment of other execution proteins. Then, the process of conjugation involving autophagy-related protein 3 (ATG3), autophagy-related protein 4 (ATG4), and autophagy-related protein 7 (ATG7) to protein light chain (LC3) is initiated, during which the LC3 protein is processed to LC3-I, LC3-II, and LC3-III [136]. Meanwhile, an autophagosome is formed from the phagophore, containing part of the cytoplasm along with proteins destined for degradation. The LC3-II form participates in the selection of transported material by binding to p62/sequestosome 1 (SQTM 1) [135,136]. This is followed by the fusion of the autophagosome with the lysosome, resulting in the formation of the autolysosome, in which the encapsulated material undergoes enzymatic digestion. The general concept of autophagosome formation is shown in Figure 4. Wang et al. reported that BA induces autophagy of SGC-7901 gastric cancer cell line, indicating a more versatile mode of action of BA [126]. This was indicated by increased levels of LC3-II and decreased expression of LC3-I and p62 [126]. A decrease in the expression levels of phosphorylated ERK and MEK proteins was also observed. This suggests that the anti-tumor effect of BA on SGC-7901 gastric cancer cells is related to the induction of cell death with autophagy and blockage of the ERK/MEK signaling pathway [126]. To circumvent cellular mechanisms of drug resistance, as well as to increase the solubility and bioavailability of betulinic acid, new formulations of BA are developed. Wang et al. compared the efficacy of free betulinic acid with the effects of betulinic acid-loaded nanoliposomes on colon cancer cells [137]. Both formulations showed significant anti-cancer effects; however, the nanoliposomal formulation showed superior anti-cancer activity in vitro and in vivo as indicated by increased cellular uptake and proliferation inhibition compared with free BA. Zhao et al. used betulinic acid nanoparticles on lung cancer cells of HKULC2, H1299, and H23 lines [138]. The results showed a decrease in proliferation in all cell lines tested. HKULC2 proved to be particularly sensitive to BA treatment, where a decrease in migratory potential, invasiveness, and metastatic capacity were also observed [138].

The first phase I/II clinical trials on BA were implemented in 2006 (NCT00346502, University of Illinois, USA, www.clinicaltrials.gov). Their objective was to evaluate the safety and efficacy of an ointment containing 20% BA in the treatment of dysplastic melanocytic nevi (DMN), which in many cases can be a precursor to melanoma. Unfortunately, due to a lack of funding, the study was suspended in 2013, and the results were not published. Another phase I clinical trial began in 2008, where BA (ALS-357) was administered as an ointment for 4 weeks to patients with metastatic melanoma. In addition to efficacy and safety, histopathological features and apoptotic markers were evaluated. Unfortunately, the results were never published. Information on the range of concentrations tested was also not provided. 

Interest has shifted from betulinic acid to its natural and synthetic derivatives, which, in addition to their anti-cancer activity, are also characterized by high cytotoxicity and better water solubility. Liebscher et al. studied the effects of BA and its derivatives, NVX-207 and B10, on melanoma cells in horses [139]. All examined compounds exhibited cytotoxic effects, promoting death through activation of the intrinsic apoptotic pathway. NVX-207 showed the highest efficacy and was then encapsulated in 2-hydroxyprolyl-β-cyclodextrin and used in in vivo studies in melanoma-affected horses. The substance was well-tolerated. The safety and efficacy of NVX-207 were also evaluated in phase I/II clinical trials in dogs with spontaneous tumors [140]. Treatment consisting of topical administration of NVX-207 and NVX-207 in combination with cisplatin resulted in a favorable response to treatment in the form of a reduction in tumor size in all subjects. The therapy was not accompanied by systemic side effects [140]. 

Despite the many benefits of BA in vitro and in vivo, clinical trials examining its anti-cancer properties seem to have stalled long ago. Clinical trials focusing on other properties of this compound, i.e., anti-anxiety and stress-reducing properties, have been undertaken (NCT03904511, University of Ottawa, Canada, www.clinicaltrials.gov). Nevertheless, there has been a recent resurgence of interest in compounds of natural origin. Nowadays, the number of studies on natural compounds are increase rapidly, including research on BA. Perhaps this the aspect of betulinic acid’s anti-cancer properties will also return in clinical trials. Examples of preclinical studies with betulinic acid and its derivatives and clinical trials are presented in Table 3.

### 4.4. Resveratrol

Resveratrol is a natural organic compound, belonging to the group of polyphenols and based on a stilbene scaffold. It is synthesized by an enzyme, stilbene synthase, as a secondary plant metabolite [146,147]. Its synthesis and accumulation usually occur in response to stress conditions, i.e., pathogen infection, mechanical damage, or unfavorable abiotic conditions. Resveratrol was first isolated in the late 1930s from the root of *Veratrum album*. Subsequently, it was also discovered in *Polygonum cuspidatum*. It is now known to occur in more than 70 plant species, including grapes and red wine, blueberries, bilberries, black currants, raspberries, peanuts, and products containing cocoa [148,149,150]. Due to the complicated and inefficient extraction process from plant tissues, alternative methods of production are used, including synthesis in recombinant yeast [151]. 

**Figure 5:**
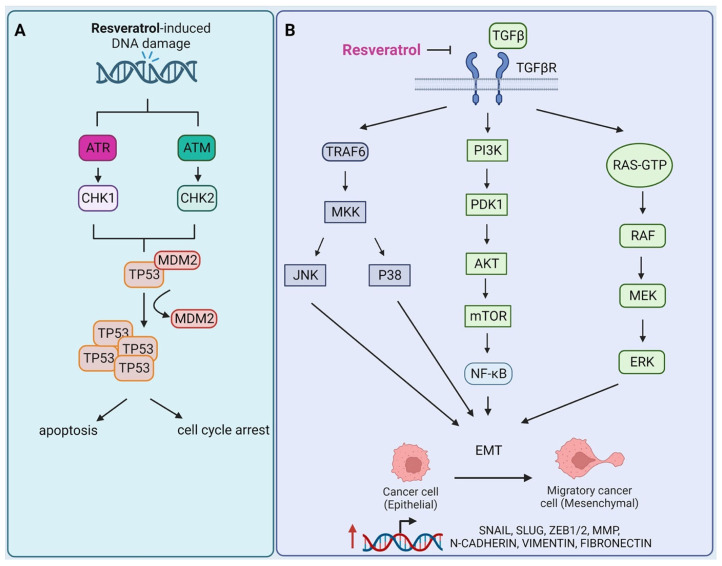
(**A**) Resveratrol can induce DNA damage, resulting in the recruitment of ataxia teleangiectasia mutated (ATM)/ataxia teleangiectasia and RAD3 related (ATR) kinases that phosphorylate checkpoint kinase 1 (CHK1)/checkpoint kinase 2 (CHK2) serine-threonine kinases. This prevents the interaction of tumor protein P53 (*TP53*) with mouse homolog of double minute 2 (MDM2), thereby protecting the *TP53* protein from degradation. *TP53* induces the expression of genes involved in cell cycle arrest and apoptosis. (**B**) Epithelial–mesenchymal transition (EMT) is initiated by many factors, including the activation of transforming growth factor β receptor (TGF-βR) by the native ligand transforming growth factor β (TGF-β). This event initiates the signaling through rat sarcoma virus-GTP (RAS-GTP)/RAF proto-oncogene serine/threonine-protein kinase (RAF)/mitogen-activated protein kinase (MEK)/extracellular signal-regulated kinase (ERK) and phosphoinositide 3-kinase (PI3K)/phosphatidylinositol kinase (PDK-1)/protein kinase B (AKT)/mammalian target of rapamycin (mTOR)/ nuclear factor kappa-light-chain-enhancer of activated B Cells (NF-κB) pathways, resulting in the expression of EMT-related factors. EMT can also be promoted by c-Jun N-terminal kinase (JNK) and p38 kinase, which are members of the mitogen-activated protein kinase (MAPK) pathway. They are activated by MAP kinase (MKK) and TNF receptor-associated factor 6 (TRAF6) with K63-linked polyubiquitin chains by TGF-β-dependent polyubiquitinylation. Resveratrol inhibits TGF-β and TRAF6, preventing EMT of cancer cells. Created with BioRender.com.

Resveratrol is a natural anti-oxidant used in the fight against free radicals and photoaging. It exists in two isomeric forms: an intractable *cis-* and a highly active *trans-* form. Conversion from *trans-* to *cis-* isomer occurs due to UV radiation and/or under high pH conditions [152]. Due to its stability, research has focused mainly on *trans-*resveratrol. Its structure features two phenol rings, and unlike, the stilbene from which it is derived, the phenyl groups at the 3’, 4’, and 5’ positions of the ring have been substituted by hydroxyl groups, which influence its anti-oxidant properties. Stivala et al. showed that the presence of the 4’ hydroxyl group is necessary for its activity, while the presence of other 3’ and 5’ enhance its biological effects [153]. In addition to anti-oxidant properties, resveratrol also exhibits anti-inflammatory and anti-cancer activities. It acts pleiotropically by modulating the activity of multiple signaling pathways, including the DDR pathway [154]. Resveratrol leads to the stimulation of the *TP53* via phosphorylation by the phosphatidylinositol 3-kinase-related kinases (PIKK) family kinases, including ataxia teleangiectasia mutated (ATM) and ataxia teleangiectasia and Rad3 related (ATR). ATM/ATR phosphorylates checkpoint kinase 1 (CHK1) and checkpoint kinase 2 (CHK2) therefore stabilize TP53 and prevent its interaction with inhibitory factor MDM2 [155] (Figure 5A). The *TP53* gene product TP53 works as a transcription factor for many target genes and is a critical tumor suppressor protein [156]. Shang et al. described the anti-inflammatory and cardioprotective properties of resveratrol in a rat model of cardiomyopathy and sepsis [157]. The authors investigated the relationship between the PI3K/AKT/mTOR signaling pathway and NF-κB activity, and the effect of resveratrol on both pathways. Rats treated with resveratrol showed inhibition of myocardial tissue damage and a decrease in the number of apoptotic cells. This was accompanied by an increase in the expression of PI3K, AKT, and mTOR proteins, with down-regulation of factors associated with the NF-κB pathway i.e., TNF-α, IL-6, IL- 1β, and toll-like receptor 4 (TLR4) [157]. Mohapatra et al. evaluated the effects of resveratrol on MCF-10A mammary gland epithelial cells transformed with cigarette smoke condensate (CSCs) in a mouse xenograft model [158]. Treatment of MCF-10A with resveratrol-caused cell death via the apoptotic pathway, which is related to resveratrol’s ability to trigger damage to DNA and affect the DNA repair processes occurring in cells. A dose-dependent decrease in the expression of the main protein components of the base excision repair (BER) pathway, including β, δ, ε polymerases, proliferating cell nuclear antigen (PCNA), and Flap endonuclease 1 (FEN-1), were demonstrated in MCF-10A, while no changes in gene expression were observed in P21-depleted MCF-10A cells. This is consistent with previous reports on the role of P21 in interference with DNA repair factors, such as DNA polymerase δ [159] and FEN-1 [158,160]. In the in vivo model, a reduction in tumor size and a decrease in the expression of anti-apoptotic markers PI3K/AKT and NF-κB were observed. The results suggest that resveratrol-induced impairment of BER activity may trigger apoptosis in a P21-dependent manner [158]. The above studies also demonstrate the dualistic nature of resveratrol, which can protect cells from apoptosis and inflammation but can also induce the death of cancer cells. Another important process involved in cancer metastasis is the EMT. In this transition, cells lose their epithelial characteristics in favor of mesenchymal characteristics. Until recently, this process was understood to include two separate cell populations and to be accompanied by a decrease in the expression of epithelial markers (E-cadherin, cytokeratins) and an increase in mesenchymal markers (N-cadherin, vimentin, fibronectin, MMPs) [161]. However, recent research suggests that EMT is gradual process and is manifested by varying cellular states and fluctuating levels of epithelial and mesenchymal markers [162,163]. Thus, cells may show intermediate or hybrid EMT features, taking into account morphological, transcriptional, and epigenetic biological aspects [161]. EMT is triggered by many factors, the most prominent of which is TGF-β, which binds to its cognate receptor transforming growth factor β receptor (TGF-βR) [164]. As a result of promoting EMT, the cell initiates the expression of mesenchymal markers and transcription factors: zinc finger protein SNAI1 (SNAIL), zinc finger protein SNAI2 (SLUG), and zinc finger E-box binding ½eobox 1/2 (ZEB1/2) [165]. During EMT, the loss of connection with the basement membrane and other cells, digestion of the extracellular matrix, and increased cell motility are observed [161]. The process of MAPK and AKT pathways activation that promotes EMT is shown in Figure 5B. Resveratrol has been shown to inhibit TGF-β-induced EMT in LoVo colon cancer cells in vitro and in vivo, accompanied by a decrease in the expression of EMT-related factors, i.e., MMPs, extracellular matrix-degrading proteins, and vascular basement membrane proteins that normally promote metastasis [166]. Li et al. have also reported that resveratrol caused a therapeutic effect by inhibiting the viability, proliferation, and EMT of prostate cancer cells by reducing the level of TNF receptor-associated factor 6 (TRAF6) [167]. Overexpression of TRAF6 may lead to increased cell migration and promote the transcription of genes encoding EMT-related proteins. TRAF6 undergoes K63-linked ubiquitination induced by TGF-β, which, unlike K48-linked ubiquitination, does not degrade the protein, but instead serves as a regulatory signal and a scaffold for the assembly of protein complexes responsible for the activation of other protein mediators [168]. Polyubiquitinylated TRAF6 is required for the activation of the tertiary kinase cascade, which activates the N-terminal kinase c-Jun (JNK) and p38. Increased activation of JNK and p38, which belong to the MAPK pathway, is accompanied by overexpression of the transcription factors SNAIL and SLUG and promotes tumor suppression and EMT transition [168]. The above examples underline the effects of resveratrol on key signaling pathways involved in the etiopathogenesis and development of cancer. Due to the plethora of publications in this review, we did not focus on a specific type of cancer, but rather on the mechanism of action. This topic was reviewed elsewhere [169]. 

Due to its high therapeutic potential and success in preclinical studies, resveratrol has moved into clinical trials. The first reports in vitro and in vivo, focusing on the pharmacokinetics and safety of the substance, showed that the concentrations used in tests are not achievable in humans [170]. The low systemic availability is most likely attributed to the compound’s rapid metabolism. Moreover, resveratrol exhibited a non-linear dose-response relationship. In phase, I clinical trial (NCT0098969, University of Michigan Comprehensive Cancer Center, Ann Arbor, Michigan, USA, www.clinicaltrials.gov), Brown et al. administered resveratrol to 40 healthy subjects at a dose of 0.5, 1, 2.5, or 5 g daily for 29 days [170]. All doses used were safe, although the two highest doses (2.5 and 5 g) reported mild gastrointestinal symptoms. The most effective inhibitory effect on the expression of plasma IGF-1 and IGFBP-3 was observed for a dose of 2.5 g/day. In contrast, at a dose of 5 g/day, the highest circulating levels of the parent agent were observed; these levels were close to the concentrations achieved in cells in vitro and were intended to cause pharmacological activity. Cai et al. compared the tissue distribution and efficacy of different doses of resveratrol ex vivo and in vivo [171]. The results showed that resveratrol at a low dose of 5 mg/day had higher efficacy compared to the dose used by Brown et al. The authors also suggested that the anti-neoplastic properties of resveratrol result from autophagy and senescence induction of cancer cells [171]. Other studies investigated resveratrol used in milligram doses; however, they failed to achieve the target IC_50_ concentration in human plasma needed for chemopreventive effects [172,173]. In an attempt to circumvent the compound’s bioavailability problem, a micronized formulation of resveratrol was used in phase I clinical trials in patients with colorectal cancer (NCT00920803, GSK Investigational Site, Leicester, Leicestershire, United Kingdom, www.clinicaltrials.gov). This allowed for a three-times higher maximum concentration compared to non-micronized resveratrol. The authors reported a pro-apoptotic effect of the compound manifested by an increase in the expression of cleaved caspase-3 [174]. Resveratrol together with other anti-oxidant compounds is being investigated for its epigenetic influence and reduction of DNA damage (NCT05306002, Institute for Social Security and Services for State Workers, Merida, Yucatán, Mexico, www. clinicaltrials.gov) and in phase II study in combination with sirolimus in patients with lymphangioleiomyomatosis (LAM) (NCT03253913, University of Cincinnati, Ohio, United States, www.clinicaltrials.gov).

The results obtained so far show that resveratrol is a thriving preventive and/or therapeutic agent against many types of cancer. Like most natural compounds, it has some limitations, like low bioavailability, poor solubility, and rapid metabolism. Work is underway to overcome the described problems with available tools, i.e., nanoparticles, biodegradable formulations, use of resveratrol derivatives or metabolites, and others [175]. Resveratrol is one of the hot topics in the field of oncology and the development of new anti-cancer agents. Examples of preclinical studies with resveratrol and clinical trials are presented in Table 4.

### 4.5. Roburic Acid

Roburic acid (RA) is a tetracyclic triterpenoid and a terpene derivative, encompassing the combination of five-carbon isoprene units. It was first isolated from oak galls, which are growths developed from injury to plant tissues caused by insects feeding on them. It was also found in the plants *Gentiana macrophylla* and *Gentiana dahurica*, used in traditional Chinese herbal medicine. The first studies on this compound showed its broad biological activity, including anti-oxidant, anti-microbial, anti-atherosclerotic, and anti-inflammatory properties [179,180,181]. 

Roburic acid and its derivatives have been categorized as inhibitors of microsomal prostaglandin E2 synthase-1 (mPGES-1), which prevent the formation of PGE2, a key mediator in inflammation and tumorigenesis [182]. Triterpenic acids, including RA, are found in acidic fractions of resin gums (frankincense extracts) of *Boswella* spp. plants. These compounds interfere with the activity of mPGES-1 and are involved in the conversion of arachidonic acid (AA) by COX-1 to PGE2 [182]. In addition, RA has also been identified as a COX-1 and COX-2 inhibitor, which may explain its anti-inflammatory properties [183]. Chen et al. showed that roburic acid reduced NO and IL-6 production in LPS-stimulated macrophages. In addition, it inhibited phosphorylation and prevented the degradation of IκBα, which acts as an inhibitor of NF-κB [179]. The biological activity of RA was associated with the increase in the expression of cytosolic P65. This constitutes evidence of inhibition of NF-κB translocation from the cytoplasm to the nucleus. In the same study, the authors also assessed the activation of the MAPK pathway, which, like NF-κB, regulates gene transcription, protein biosynthesis, and the processes of cell division, differentiation, and survival. RA inhibited the phosphorylation of two major effector kinases of this pathway: JNK and p38. No effect on ERK1/2 kinases was observed [179]. In another study, the authors showed that inhibition of NF-κB signaling occurs through direct interaction of RA with TNF-α, which prevents the native ligand from binding the target tumor necrosis factor receptor 1 (TNF-R1) [180]. Changes in the expression of cell cycle marker proteins after RA treatment of HCT-116 and HCT-15 cells were also studied. The regulation of the cell cycle takes place with the participation of cyclin-dependent kinases (CDKs), which, when activated as a result of association with cyclins, stimulate the progression of the cell cycle. For instance, cyclins D and E promote transition to the G1 phase (D-CDK4/6; E-CDK2), cyclin B1 to M phase (B-CDK1), and cyclin A to S and G2 phase (A-CDK2) [184]. Their synthesis in cell takes place *de novo*, and the concentrations of specific cyclins change as the cell cycle progresses; an increase in the concentration leads to the activation of the partner CDK and is followed by a decrease due to cyclin proteasomal degradation, which restores CDK to an inactive state. Disturbances in the expression of cyclins may lead to the development of neoplasia. It was shown that RA exerted an anti-proliferative effect by reducing the expression of cyclins B, D, and E, which led to cell cycle arrest in the G0/G1 phase [180]. In addition, it reduced the expression of the c-MYC oncoprotein, which also promotes the transition of cells from the G1 to the S phase. In the same study, the level of apoptosis regulatory proteins of the BCL-2 family was also evaluated. These proteins are divided into two subgroups that promote or block apoptotic signals by interacting with other proteins involved in the process of cell death. Pro-apoptotic proteins composed of a single BH3 domain (BH3 interacting-domain death agonist (BID); BCL-2 interacting mediator of cell death (BIM); P53 upregulated modulator of apoptosis (PUMA); phorbol-12-myristate-13-acetate-induced protein 1 (NOXA)) play the role of activators, receiving and transmitting the apoptotic signal to pro-apoptotic proteins of multi-domain proteins with an effector function (BAX and BCL-2 homologous antagonist killer (BAK)) [185]. These proteins cause changes in the permeability of the outer mitochondrial membrane, which leads to the release of cytochrome C and the formation of the apoptosome, which is needed for autocatalytic maturation and activation of caspases. Caspases occur in the form of zymogens that undergo proteolytic activation [186]. The role of the resulting “caspase cascade” is to initiate events related to apoptotic death. HCT-116 and HCT-115 cells treated with RA showed a decrease in anti-apoptotic proteins, i.e., B-cell lymphoma 2 (BCL-2), BCL-xL, X-linked inhibitor of apoptosis protein (XIAP), myeloid leukemia cell 1 (MCL-1), and survivin. Moreover, RA promoted proteolytic cleavage of effector caspases -3 and -7, leading to their activation [180]. The anti-inflammatory and anti-oxidant properties of RA were also investigated by Wang et al., where they caused attenuation of receptor activator of nuclear factor Kappa-B ligand (RANKL)-induced osteoclastogenesis. RA not only reduced the number of osteoclasts, but also reduced hydroxyapatite resorption and calcium oscillation [181]. Moreover, it down-regulated TRAF6 expression and inhibited related signaling cascades, including ERK1/2 phosphorylation and IκBa degradation. It also promoted the activation of the nuclear factor-erythroid 2 related factor 2 (NRF2)/ kelch-like ECH-associated protein 1 (KEAP1)/anti-oxidant response element (ARE) pathway, which is responsible for the production of anti-oxidants in opposition to reactive oxygen species (ROS) production promoting TRAF6 effects [181]. With promising anti-inflammatory and pro-apoptotic properties, roburic acid has emerged as an interesting candidate anti-cancer agent. Wang et al. tested RA in combination with docetaxel and a proton pump inhibitor esomeprazole (ESOM) on human PC3 prostate cancer cells. The authors noted the benefits of the synergistic action of the two substances manifested by inhibition of tumor cell viability, suppression of the NF-κB pathway, and induction of apoptosis. In addition, a reduced tendency to form spheres was observed, indicating inhibition of the ability of cancer stem cells to self-renew [187]. In addition to its important anti-inflammatory and anti-tumor effects, roburic acid has also shown therapeutic effects in Alzheimer’s disease due to its ability to reduce the levels of neurotoxic Aβ peptide accumulation in the brain by breaking down β/γ secretase complexes without inhibiting secretase’s proteolytic activities [188]. Examples of preclinical studies with roburic acid are presented in Table 5.

Roburic acid is one of the latest discovered and the poorest studied substances of natural origin, which, in addition to its strong anti-inflammatory properties, also exhibits anti-tumor activity. Hence, it can be considered a stand-alone or adjunctive therapeutic agent for further studies. This topic, however, requires further research. 

### 4.6. Other Substances

In addition to the compounds discussed here, several other substances are being studied as anti-cancer agents. Of note are natural plant polyphenols, such as epigallocatechin-3-gallus (EGCG) that are found in green tea extracts. EGCG has proven anti-cancer, anti-oxidant, and anti-inflammatory properties, providing many chemotherapeutic as well as chemopreventive benefits [189,190]. EGCG disrupted the growth of SH-SY5Y immature neuroblastoma by targeting the dual specificity tyrosine-phosphorylation-regulated kinase 1A (DYRK1A) in vitro [191]. It has also been reported to play a crucial role in the promotion of apoptotic cell death through inhibition of the epidermal growth factor receptor (EGFR), signal transducer and activator of transcription 3 (STAT3), and NF-κB transcription factor pathways in breast cancer cells and head and neck squamous cell carcinoma cells [192,193]. In vivo, it demonstrated inhibition of tumor growth, accompanied by suppression of metastasis and prolongation of survival time in a 4T1 mouse model of breast cancer [194]. Among patients with advanced precancerous lesions of the head and neck, treatment with the combination of EGCG and erlotinib was well-tolerated and had a high pathological response rate (NCT01116336, www.clinicaltrials.gov) [195]. Phase I clinical trials with EGCG for colon cancer patients (NCT02891538, www.clinicaltrials.gov), for cirrhosis-free liver cancer participants (NCT03278925, www.clinicaltrials.gov), and even studies of the role of EGCG for severe acute respiratory syndrome coronavirus 2 (SARS-CoV-2)-infected cancer patients (NCT05758571, www.clinicaltrials.gov) are currently underway (recruitment phase). The clinical trial using EGCG, quercetin, zinc, and metformin as adjuvant compounds in add-on therapy for early metastatic breast cancer and triple-negative breast cancer is also planned (NCT05680662).

Other polyphenols include the calebin A and curcumin components of turmeric, which have shown anti-inflammatory and anti-tumor properties, particularly in colorectal cancer [196,197]. These substances can re-sensitize resistant colorectal cancer cells to commonly used chemotherapeutics. Furthermore, they can regulate growth factors, transcription factors, inflammatory cytokines, and proteins of the BCL-2 family [198,199]. The potential of curcumin has also been tested in conjugation with antibodies and adjuvants to increase the selectivity and improve the biological activity of the compound in future clinical trials [200,201]. When combined with conventional anti-cancer therapy, the benefits of multidirectional activity and reduced toxicity have been observed. Several reviews have been published describing the effect of curcumin in preclinical models and clinical studies [196,197,202].

Also, other natural substances belonging to the group of alkaloids (berberine [203], nephrine [204]), xanthonoids (kaempferol [205]), phenols (capsaicin [206]), and flavonoids (salvigenin [207]) show interesting chemoprotective and chemotherapeutic properties, which require further research. Due to the vastness of the topic, this paper does not discuss all of them.

## 5. Conclusions and Future Prospects

Plants are a rich source of substances that have been used in herbalism and natural medicine for centuries. Many of them constitute the blueprint for the development of synthetic compounds for the treatment of cancer. The most commonly used natural, plant-derived anti-cancer agents include paclitaxel, *vinca* alkaloids, camptothecin derivatives, and podophyllotoxins. An important advantage of these compounds is their multidirectional action, manifested by modulation of the activity of key signaling pathways and multiple molecular targets involved in cancer cell proliferation, migration, and apoptosis. Increased research into the abnormalities of cellular signaling pathways, as well as an understanding of the function and cooperation of oncogenes and anti-oncogenes, boosts the search for new substances and development of analogs with enhanced anti-cancer activity, as well as refined safety profile circumventing problems related to delivery, metabolism, and bioavailability of natural compounds. The natural compounds described in this review are currently being tested in both clinical and preclinical studies and are potential candidates for clinical use. However, despite the high level of interest, the complex nature of cellular response triggered following their use means that they require comprehensive research. 

Each of the compounds described in this review that has entered the clinical trial phase has shown side effects or toxicities that limit its use. Among the main side effects of paclitaxel are myalgia, arthralgia, nausea, diarrhea, mucositis, hypotension, bradycardia, and others [46]. The described cases of neutropenia necessitated changes in the dosing schedule and shortened the drug’s infusion time. The appearance of thrombocytopenia or anemia has been observed with less severity [208]. The most commonly reported infections during paclitaxel treatment include urinary tract and upper respiratory tract infections, as well as episodic pneumonia and peritonitis. Neurotoxic events, which result in numbness, autonomic system dysfunction, and movement disorders, are also observed in a dose- and time-dependent manner [209]. Severe toxicities occur in 5–10% of patients treated with paclitaxel [210]. Palliative therapy options include dose reduction and discontinuation of treatment. Hypersensitivity to paclitaxel can also be minimized with corticosteroids and antihistamines [46]. Another of the drugs mentioned in this review, irinotecan, is known mainly for gastrointestinal complaints and neutropenia, limiting the dose of the drug [211]. Both monotherapy and the combination regimen of 5-fluorouracil, leucovorin, and irinotecan (FOLFIRI) show severe diarrhea and neutropenia in approximately 50% of patients [212]. Their duration is correlated with the length of drug exposure. Attempts have been made to reduce toxicity using cyclosporine and phenobarbital to modulate the pharmacokinetics of irinotecan, with positive results in patients [213]. Other studies have suggested a reduction in severe diarrhea after combining irinotecan with antibiotics or probiotics [211,214]. Similarly, the severity of side effects depends on the dose and duration of exposure to resveratrol. Mild and short-term side effects include gastrointestinal complaints, headache, and rash, which occur at doses greater than 2.5 g/day [170,215]. At doses greater than 5 g/day, patients have been found to exhibit signs of nephrotoxicity [216]. Cases of renal failure were observed only with monotherapy, while this severe side effect is scarce when administered in combination with bortezomib [175]. There are still few results from human studies of resveratrol, so both the distribution in tissues and the observed toxicity leave many open questions. There is a lack of information on monotherapy and combination therapies based on resveratrol or betulinic acid.

The described examples of drug toxicity and side effects can be overcome with specific combinations of substances, either through their delivery or the use of nontoxic prodrugs metabolized to their active form. In 2022, Nasibullin et al. published the results of a project in which they designed a retrosynthetic prodrug that was converted using artificial metalloenzymes [217]. The authors put emphasis on identifying useful chemical reactions that lead to the formation of the pharmacophore, and thus designed a naphthylclombretastatin-based prodrug that forms highly active cytostatic agents through sequential ring-closing metathesis and aromatization. Based on this mechanism, the substance showed excellent anti-cancer activity in vivo [217]. The described methodology may allow for the retesting of drugs whose use has been restricted due to high toxicity. Drug efficacy and bioavailability can also be improved by designing and synthesizing new derivatives with an improved pharmacokinetic profile. Currently, drug design is refined with the use of bioinformatics and cheminformatics. Computer-aided drug design involves identifying potential targets, searching databases for drug candidates, further optimizing candidate compounds, and assessing their potential toxicity. Following these analyses, the compounds are subjected to in vitro and in vivo experiments. Natural substances or plant-based compounds can provide excellent examples of lead structures that can be used as a starting scaffold for the development of new structures with improved physicochemical parameters. Current experimental approaches involve the review and re-testing of currently known substances for new indications in a process of drug-repurposing. Natural substances are particularly useful for this given their wide biological activity and the multiple molecular pathways they target. Both toxicity and bioavailability limitations can be addressed through appropriately selected carriers, delivery systems, and pro-drug design. In addition, there is an increased focus on personalizing each patient’s treatment strategy based on the results of a screening to identify genes that confer resistance to specific chemotherapy.

## Figures and Tables

**Table 1:** Preclinical and clinical studies with paclitaxel and its derivatives in monotherapy and combination therapies.

Agent	Type of Study	Eligible Disease Site(s) and Organism	Combinational Agent	Ref.
Paclitaxel Nab-paclitaxel	In vivo	Mouse model of B16F10 melanoma	Albumin-coated nanocrystal formulation of Paclitaxel and Abraxane (Nab-paclitaxel)	[78]
Docetaxel	In vivo	Rats’ circulation system	Docetaxel nanocrystal-loaded micelles	[79]
Cabazitaxel	In vitro and in vivo	Mouse xenograft model PC346C-DCCK	Cabazitaxel and enzalutamide	[80]
Agent	Phase of Clinical Trial	Eligible Disease Site(s)	Combinational Agent	Nct Clinical Trial Number
Paclitaxel	Phase 1	Ovarian cancer	Fostamatinib and Paclitaxel	NCT03246074
Paclitaxel	Phase 2	Lung cancer	Carboplatin, Paclitaxel, and AZD1775	NCT02513563
Paclitaxel	Phase 1/2	Gastric cancer	Variltinib and Paclitaxel	NCT05400915
Paclitaxel	Phase 2	Breast cancer	Cisplatin vs. Paclitaxel	NCT01982448
Paclitaxel	Phase 1	Breast neoplasms	Cirmtuzumab and Paclitaxel	NCT02776917
Nab-paclitaxel	Phase 1/2	Pancreatic cancer	Gemcitabine, Nab-paclitaxel, and Paricalcitol	NCT03520790
Nab-paclitaxel	Phase 1	Breast cancer	Pembrolizumab and Nab-paclitaxel	NCT02999477
Nab-paclitaxel Paclitaxel	Phase 3	Breast cancer	Abraxane (Nab-paclitaxel) and Paclitaxel	NCT01822314
Nab-paclitaxel	Phase 2	Breast cancer	Mifepristone and Nab-paclitaxel	NCT02788981
Nab-paclitaxel	Phase 2	Breast cancer	Single agent	NCT04192331
Docetaxel	Phase 3	Prostate cancer	Single agent	NCT00653848
Docetaxel	Phase 2	Breast cancer	Docetaxel and Carboplatin	NCT02547987
Cabazitaxel	Phase 2	Prostate cancer	Abiraterone acetate, Cabazitaxel, and Prednisone	NCT02218606
Cabazitaxel	Phase 3	Adenocarcinoma of prostate/progression of prostate cancer	Cabazitaxel and radiotherapy	NCT01952223

**Table 2:** Preclinical and clinical studies with irinotecan and its modification in monotherapy and combination therapies.

Agent	Type of Study	Eligible Disease Site(s) and Organism	Combinational Agent	Ref.
Irinotecan	In vitro and in vivo	Mouse H22 tumor model	Irinotecan-loaded PLGA microspheres	[107]
Irinotecan	In vivo	Rabbit VX2 liver tumor model	Irinotecan-loaded QuadraSphere microspheres	[108]
Irinotecan	In vitro and in vivo	Wistar rats and potentially colon cancer	Irinotecan-loaded solid lipid nanoparticles	[109]
Agent	Phase of Clinical Trial	Eligible Disease site(s)	Combinational Agent	NCT Clinical Trial Number
Irinotecan	Phase 1/2	Small cell lung cancer	DS.-3201b and Irinotecan	NCT03879798
Irinotecan	Phase 3	Small cell lung cancer	Irinotecan liposome injection vs. Topotecan	NCT03088813
Nanoliposomal Irinotecan	Phase 1	Refractory solid tumors	Nanoliposomal Irinotecan and TAS-102	NCT03810742
Nanoliposomal Irinotecan	Phase 1/2	Biliary tract cancer	Nivolumab plus nanoliposomal Irinotecan, 5-Fluorouracil, and Leucovorin	NCT03785873
Irinotecan Hydrochloride	Phase 1	Metastatic colorectal carcinoma	Utomilumab, catuximab, and irinotecan hydrochloride	NCT03290937

**Table 3:** Preclinical and clinical studies with betulinic acid and its derivatives.

Agent	Type of Study	Eligible Disease Site(s) and Organism	Combinational Agent	Ref.
Betulinic acid	In vitro and in vivo	Murine models with TNBS- and DSS-induced inflammatory bowel disease	Hydroxamate of Betulinic Acid	[141]
Betulinic acid	In vitro and in vivo	Mouse model with colorectal cancer	Betulinic Acid-loaded liposomes coated with Eudragit S100	[142]
Betulinic acid analogue (2c)	In vitro and in vivo	Mice and rats with colorectal cancer	Nanoencapsulated betulinic acid analogue	[143]
Betulinyl-bis-sulfamate NVX-207	In vitro	Equine sarcoid cells, equine malignant melanoma, and fibroblasts	Betulinyl-bis-sulfamate NVX-207	[144]
Betulinic acid NVX-207	In vivo	Horses with melanocytic tumors	Betulinic acid and NVX-207	[145]
Agent	Phase of Clinical Trial	Eligible Disease Site(s)	Combinational Agent	NCT Clinical Trial Number
Betulinic acid	Phase 1 (unknown)	Melanoma	ALS-357 (Betulinic Acid)	NCT00701987
Betulinic acid	Phase 1/2 (withdrawn)	Dysplastic Nevus Syndrome	20% Betulinic Acid Ointment	NCT00346502

**Table 4:** Preclinical and clinical studies with resveratrol.

Agent	Type of Study	Eligible Disease Site(s) and Organism	Combinational Agent	Ref.
Resveratrol	In vivo	Athymic BALB/C nude mice injected with HeLa cells (cervical cancer)	Single agent	[176]
Resveratrol	In vivo	Mouse xenograft tumor model cervical cancer	Hydroxypropyl-β-cyclodextrin-complexed Resveratrol	[177]
Resveratrol	In vitro and in vivo	Mouse xenograft tumor model cervical cancer	Single agent	[178]
Agent	Phase of Clinical Trial	Eligible Disease Site(s)	Combinational Agent	NCT Clinical Trial Number
Resveratrol	Phase 1 (completed)	Colon cancer	Single agent	NCT00256334
Resveratrol	Phase 1 (completed)	Colorectal cancer	Single agent	NCT00433576
Resveratrol	Phase 1 (completed)	Healthy participants	Single agent	NCT00098969
Resveratrol (SRT501)	Phase 1 (completed)	Neoplasms, colorectal	Single agent	NCT00920803
Resveratrol	Phase 2 (completed)	Lymphangioleiomyomatosis	Resveratrol and Sirolimus	NCT03253913
Resveratrol	Not applicable (active)	HBOC Syndrome, nutrition therapy	Resveratrol with anti-oxidant therapy dietary components	NCT05306002

**Table 5:** Preclinical studies with roburic acid.

Agent	Type of Study	Eligible Disease Site(s) and Organism	Combinational Agent	Ref.
Roburic acid	In vitro and in vivo	Nude mouse xenograft tumor model of colorectal cancer	Single agent	[180]
Roburic acid	In vitro and in vivo	Ovariectomized mice model for treating osteoporosis	Single agent	[181]
Roburic acid	In vitro	Prostate cancer cells	Roburic acid, Docetaxel, and Esomeprazole	[187]
Roburic acid	In vitro	RAW264.7 macrophage cells	Single agent	[179]

## Data Availability

No data was generated during writing this manuscript.

## Abbreviations

3LL	Lewis lung cancer
AA	Arachidonic acid
AKT	Protein kinase B
AMPK	5′ AMP-activated protein kinase
ARE	Anti-oxidant response element
ATG13	Autophagy-related protein 13
ATG3	Autophagy-related protein 3
ATG4	Autophagy-related protein 4
ATG5	Autophagy-related protein 5
ATG7	Autophagy-related protein 7
ATG10	Autophagy-related protein 10
ATG12	Autophagy-related protein 12
ATG16	Autophagy-related protein 16
ATM	Ataxia teleangiectasia mutated
ATR	Ataxia teleangiectasia and Rad3 related
BA	Betulinic acid
BAD	BCL-2-associated death promoter
BAK	BCL-2 homologous antagonist killer
BAX	BCL-2-associated X protein
BCL-2	B-cell lymphoma 2
BCL-XL	B-cell lymphoma-extra large
BER	Base excision repair
BID	BH3 interacting-domain death agonist
BIM	BCL-2 interacting mediator of cell death
*BRCA1*	Breast cancer type 1 susceptibility protein
*BRCA2*	Breast cancer type 2 susceptibility protein
CDKs	Cyclin-dependent kinases
CHK1	Checkpoint kinases 1
CHK2	Checkpoint kinases 2
COX	Cyclooxygenase
CPT-11	Irinotecan
CSCs	Cigarette smoke condensate
DDR	DNA damage response
DSB	Double-strand breaks
DYRK1A	Dual specificity tyrosine-phosphorylation-regulated kinase 1A
ECM	Extracellular matrix
EGCG	Epigallocatechin-3-gallus
EGFR	Epidermal growth factor receptor
EMT	Epithelial-mesenchymal transition
ERK	Extracellular signal-regulated kinase
ESOM	Esomeprazole
FDA	Federal drug administration
FEN1	Flap endonuclease 1
FOXO	Fork head box O
FUBP1	Far upstream element binding protein 1
GTP	Guanosine-5′-triphosphate
IGF-1	Insulin-like growth factor-1
IGFBP-3	Insulin-like growth factor binding protein 3
IKKβ	IκB kinase
ILs	Interleukins
IκB	B-cells inhibitor
JNK	c-Jun N-terminal kinase
KEAP1	Kelch-like ECH-associated protein 1
LAM	Lymphangioleiomyomatosis
LC3	Protein light chain
LPS	Lipopolysaccharide
MAPK	Mitogen-activated protein kinase
MCL-1	Myeloid leukemia cell 1
MDM2	Mouse homolog of double minute 2
MDR	Multidrug resistance
MEK	Mitogen-activated protein kinase
MKK	MAP kinase
MLST8	mTOR associated protein LST8 homolog
MiMP	Mitochondrial membrane permeability
MMP-9	Matrix metalloproteinase-9
mPGES-1	Microsomal prostaglandin E2 synthase-1
mTOR	Mammalian target of rapamycin
mTORC1	Mammalian target of rapamycin complex 1
NF-κB	Nuclear factor kappa-light-chain-enhancer of activated B cells
NK	Natural killer
NO	Nitric oxide
NOXA	Phorbol-12-myristate-13-acetate-induced protein 1
NRE	NF-κB response elements
NRF2	Nuclear factor-erythroid 2 related factor 2
NSCLC	Non-small cell lung cancer
PCNA	Proliferating cell nuclear antigen
PD-1	Programmed death receptor
PD-L2	Programmed death receptor-ligand 2
PGE2	Prostaglandin E2
PI3K	Phosphoinositide 3-kinase
PIKK	Phosphatidylinositol 3-kinase-related kinases
PIP2	Phosphatidylinositol 4,5-biphosphate
PIP3	Phosphatidylinositol (3,4,5)-triphosphate
PUMA	P53 upregulated modulator of apoptosis
RA	Roburic acid
RAF	RAF proto-oncogene serine/threonine-protein kinase
RANKL	Receptor activator of nuclear factor kappa-B ligand
RAS	Rat sarcoma virus
ROS	Reactive oxygen species
RTKs	Receptor tyrosine kinases
SARS-CoV-2	Severe acute respiratory syndrome coronavirus 2
SLUG	Zinc finger protein SNAI2
SN38	7-ethyl-10-hydroxycamptothecin
SN38-G	SN38-glucuronide
SNAIL	Zinc finger protein SNAI1
SP1	Transcription factor Sp1
SP3	Transcription factor Sp3
SQTM	Sequestosome 1
STAT3	Signal transducer and activator of transcription 3
TGF-β	Transforming growth factor β
TGF-βR	Transforming growth factor β receptor
TLR4	Toll-like receptor 4
TME	Tumor microenvironment
TNF-R1	Tumor necrosis factor receptor 1
TNF-α	Tumor necrosis factor α
*TP53*	Tumor protein P53
TRAF6	TNF receptor-associated factor 6
UGT	Uridine 5′-diphosphate-glucuronosyltransferase
ULK1	Unc-51 like autophagy activating kinase
VEGF	Vascular endothelial growth factor
VPS34	Vesicular protein sorting kinase 34
XIAP	X-linked inhibitor of apoptosis protein
ZEB1/2	Zinc finger E-box binding homeobox 1/2
βG	β-glucuronidase
